# Robotic-assisted renal autotransplant as a novel treatment option for nutcracker syndrome

**DOI:** 10.1093/jscr/rjab580

**Published:** 2021-12-24

**Authors:** Lacy Harkrader, Yahya Alwatari, Imai Daisuke, Johanna Christanson, Aamir Khan, Chandra Bhati

**Affiliations:** Department of Surgery, Division of Transplant Surgery, Virginia Commonwealth University Richmond, Richmond, VA, USA; Department of Surgery, Division of Transplant Surgery, Virginia Commonwealth University Richmond, Richmond, VA, USA; Department of Surgery, Division of Transplant Surgery, Virginia Commonwealth University Richmond, Richmond, VA, USA; Department of Surgery, Division of Transplant Surgery, Virginia Commonwealth University Richmond, Richmond, VA, USA; Department of Surgery, Division of Transplant Surgery, Virginia Commonwealth University Richmond, Richmond, VA, USA; Department of Surgery, Division of Transplant Surgery, Virginia Commonwealth University Richmond, Richmond, VA, USA

## Abstract

Nutcracker syndrome can present with various disabling symptoms. To our knowledge, there are no reports that describe a robotic-assisted approach in its management. We present a patient who underwent robotic-assisted nephrectomy after the failure of conservative management of nutcracker syndrome and a second patient who underwent robotic-assisted nephrectomy with autotransplant. Surgery and immediate post-op courses were uncomplicated. Robotic-assisted nephrectomy with or without autotransplant can be a feasible, minimally invasive option for select patients with nutcracker syndrome.

## INTRODUCTION

Nutcracker syndrome (NCS) is a rare phenomenon that results from left renal vein (LRV) compression between the superior mesenteric artery (SMA) and the abdominal aorta [1]. NCS can present with venous congestion symptoms, most commonly including hematuria, flank pain, orthostatic proteinuria and fatigue [1, 2]. Diagnosis requires evaluation of venous flow using computed tomography (CT) angiogram, magnetic resonance angiography, doppler studies or intravascular ultrasound (IVUS) measurement of intravascular pressure [2]. A consensus on the ideal treatment of this rare syndrome has not been reached because of inconsistent symptom relief in reported management [2]. Treatment is challenging, requiring a multidisciplinary approach. Recognized options include stenting the LRV, inferior transposition of the LRV, nephrectomy or autotransplantation of the kidney into the iliac fossa [2].

Robotic-assisted surgical techniques revolutionized the field of transplant surgery [3, 4]. Minimally invasive surgery has several benefits compared to an open approach including shorter hospital stay, less pain, earlier recovery and more rapid return to daily activities [5, 6]. Therefore, using robotics for renal autotransplant has promise as a novel treatment for patients suffering from NCS. We describe two patients with diagnosed NCS who elected to undergo robotic-assisted nephrectomy, one for donation and one with autotransplant.

## CASE PRESENTATIONS

### Case #1

A 49-year-old Caucasian female with NCS, postural orthostatic tachycardia syndrome (POTS), Ehlers–Danlos syndrome (EDS), neurogenic bladder requiring self-catheterization, anxiety, and depression presented for treatment of worsening left flank pain and gross hematuria. She previously received an LRV transposition, without relief of symptoms. She underwent LRV stenting, which also failed to resolve her symptoms. She was then evaluated for a possible nephrectomy. She elected to donate her kidney as she was concerned about potential continued difficulty if she retained her kidney. Both the Selection Committee and Ethics Committee at our institution approved the donation. A robotic-assisted living donor left nephrectomy to an emotionally-related recipient was completed without immediate complications.

She was discharged on postoperative Day 2 after an unremarkable postoperative course. Her baseline creatinine was 0.75–0.85 mg/dl, at discharge was 1.1 mg/dl and had remained stable at 1-year follow-up. The patient’s flank pain and hematuria mostly resolved, but she continued to have intermittent urinary frequency, tachycardia and near-syncopal episodes secondary to underlying POTS.

### Case #2

A 22-year-old Caucasian female with history of nephrolithiasis and anxiety was diagnosed with NCS before referral to our institution. She reported intermittent, severe flank pain radiating to her back, episodes of rapid weight loss, alternating postprandial diarrhea and constipation, dizziness and macrohematuria with clots. Her symptoms were present for several years and exacerbated by exercise and food intake. CT demonstrated narrowing in the LRV where it passes between the aorta and SMA, as well as dilatation of the proximal LRV. A mesenteric duplex scan revealed compression of the LRV, as demonstrated by an increase in venous flow velocity from 22 to 131 cm/s at the compression site.

**Figure 1:**
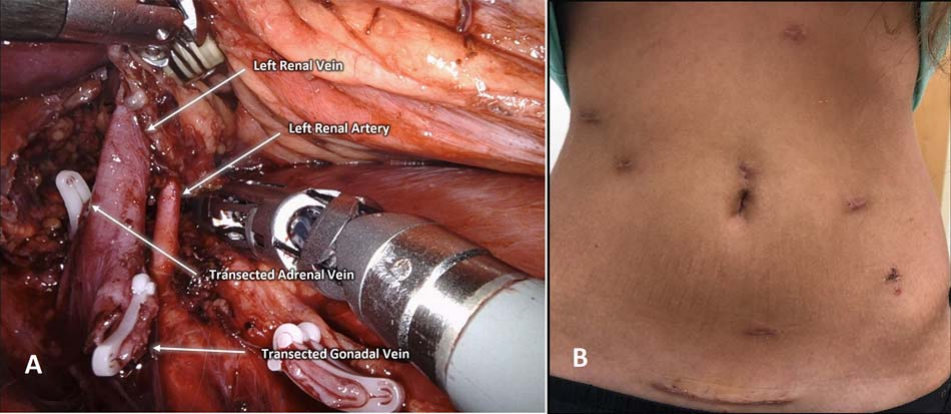
(**A**) Demonstrates robotic-assisted left nephrectomy. (**B**) Surgical incisions 10-day postoperatively.

**Figure 2:**
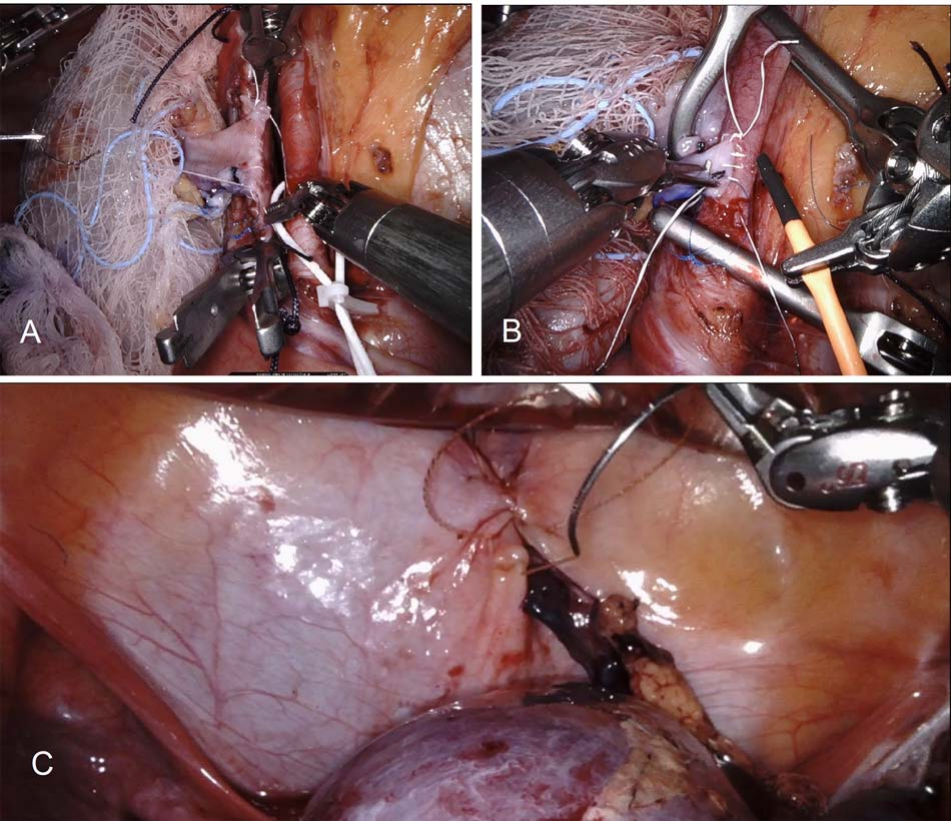
Demonstrates robotic-assisted autotransplant. (**A**) LRV to left external iliac vein anastomosis. (**B**) Left renal artery to left external iliac artery anastomosis. (**C**) Ureteral anastomosis with well-perfused kidney.

This patient elected to undergo robotic-assisted left renal autotransplantation. She underwent robotic-assisted native left nephrectomy (Fig. 1A), with back table preparation of the kidney before robotic autotransplantation into the right iliac fossa. Surgery was performed using standard kidney transplant technique. In brief, the patient is placed in Trendelenburg position (15–20-degree) to facilitate better intraoperative exposure. Four robotic ports and one 12-mm assistant port are used. A GelPoint® (Applied Medical Inc., Rancho Santa Margarita, CA) is placed by creating a 7–9-cm long midline incision involving the umbilicus. An 8-mm robotic trocar is then placed through the Gelpoint and a 30° robotic endoscope is inserted into this port. Two 8-mm robotic trocars are inserted on either side of the Gelpoint in the midaxillary line and one 8-mm port is placed near the left anterior superior iliac spine. A 12-mm assistant port is placed in the right hypochondriac region. A large peritoneal flap is created for retroperitonization of the kidney after reperfusion. The external iliac artery and vein are prepared for vascular anastomosis by dividing lymphatic tissue. The kidney is placed inside the abdominal cavity using an ice jacket via GelPoint. Bulldog clamps are used to control vessels. Polytetrafluoroethylene (PTFE) sutures are used for arterial and venous anastomosis because of their minimal suture memory (Fig. 2A and B). The ureteric anastomosis is created over a ureteric stent that is inserted into the ureter/bladder without redocking (Fig. 2C). The fashioned peritoneal flaps are used to reposition the graft in the extraperitoneal plane.

After an uneventful immediate postoperative course, she was discharged on post-op day five. At 8-month follow-up, the patient’s pain resolved with no more intake of opioids, her GI symptoms significantly improved and her creatinine remained stable at 0.82 mg/dl. Figure 1B shows her surgical incisions at 6-week follow-up.

## DISCUSSION

Symptoms caused by pathologic LRV compression and venous backup was first discussed in literature in the 1950s and was a recognized diagnosis by the 1970s [6]. Most frequently, patients experience hematuria and flank or pelvic pain thought to be related to venous backup into the left kidney and in preceding veins [6]. Patients who suffer from NCS often have multiple other associated medical problems including May–Thurner syndrome, pelvic congestion syndrome, POTS, EDS and coagulation disorders [2].

Our two patients had differing symptoms from each other. The first experienced classic symptoms of NCS and had comorbid POTS and EDS. Our second patient had classic features of macrohematuria and flank pain, but uncharacteristic alternating postprandial diarrhea and constipation. LRV compression has not previously been shown to cause GI symptoms. Decreased blood flow to the SMA due to counter-pressure from the LRV could explain these symptoms, however her mesenteric scan showed sufficient flow to the SMA. GI specialists originally diagnosed her with irritable bowel syndrome, but later, when scans showed LVR compression, determined her symptoms were more consistent with NCS. Post-autotransplant, her GI symptoms resolved. It remains unknown whether the GI symptoms were related to abnormal perfusion, venous congestion or a separate process.

Autotransplantation is a long-recognized treatment option for NCS [1–5]. Relocating the LRV prevents compression by a tight SMA-abdominal aortic angle. Although many patients undergo less-invasive options first [4], one report suggests minimally invasive autotransplantation is an appropriate first-line treatment that hastens symptomatic improvement compared to conservative interventions [4]. Our first patient underwent renal stenting and transposition but were unsuccessful at resolving her symptoms. The second patient elected to proceed directly to autotransplant as a definitive therapy.

Robotic-assisted renal transplant is reported to be advantageous to open surgery [6] due to improved cosmetic results, decreased hospital stay duration [7], improved pain outcomes and fewer surgical site infections [8–10]. Our two patients appeared to experience these positive outcomes.

## CONCLUSION

In this report, we demonstrate that robotic-assisted nephrectomy with or without autotransplant can feasibly and safely treat NCS. Both patients had symptomatic improvement with excellent renal function preservation. Larger-scale prospective studies are required to assess the efficacy and long-term outcomes of robotic-assisted surgery in the management of NCS.

## AUTHORS’ CONTRIBUTIONS

All authors designed the study, acquired the data, drafted and critically revised the manuscript, and provided approval of the final manuscript draft.
